# Improving brightness and photostability of green and red fluorescent proteins for live cell imaging and FRET reporting

**DOI:** 10.1038/srep20889

**Published:** 2016-02-16

**Authors:** Bryce T. Bajar, Emily S. Wang, Amy J. Lam, Bongjae B. Kim, Conor L. Jacobs, Elizabeth S. Howe, Michael W. Davidson, Michael Z. Lin, Jun Chu

**Affiliations:** 1 Department of Bioengineering, Stanford University, Stanford, CA, 94305, USA; 2 Department of Pediatrics, Stanford University, Stanford, CA, 94305, USA; 3 Department of Biology, Stanford University, Stanford, CA, 94305, USA; 4 National High Magnetic Field Laboratory, Tallahasee, FL, USA; 5 Department of Neurobiology, Stanford University, Stanford, CA 94305, USA

## Abstract

Many genetically encoded biosensors use Förster resonance energy transfer (FRET) to dynamically report biomolecular activities. While pairs of cyan and yellow fluorescent proteins (FPs) are most commonly used as FRET partner fluorophores, respectively, green and red FPs offer distinct advantages for FRET, such as greater spectral separation, less phototoxicity, and lower autofluorescence. We previously developed the green-red FRET pair Clover and mRuby2, which improves responsiveness in intramolecular FRET reporters with different designs. Here we report the engineering of brighter and more photostable variants, mClover3 and mRuby3. mClover3 improves photostability by 60% and mRuby3 by 200% over the previous generation of fluorophores. Notably, mRuby3 is also 35% brighter than mRuby2, making it both the brightest and most photostable monomeric red FP yet characterized. Furthermore, we developed a standardized methodology for assessing FP performance in mammalian cells as stand-alone markers and as FRET partners. We found that mClover3 or mRuby3 expression in mammalian cells provides the highest fluorescence signals of all jellyfish GFP or coral RFP derivatives, respectively. Finally, using mClover3 and mRuby3, we engineered an improved version of the CaMKIIα reporter Camuiα with a larger response amplitude.

Genetically-encoded FRET biosensors have been widely adopted to monitor biomolecular dynamics in single mammalian cells. FRET reporters have been used to track kinase activity, intracellular ion concentrations, membrane voltage, protein-protein interactions, and protein conformation changes, among other applications^1^,^2^,^3^,^4^,^5^,^6^. In FRET biosensors, cyan fluorescent proteins (CFP) and yellow fluorescent proteins (YFP) are commonly used as donors and receptors, respectively. However, CFP-YFP pairs exhibit undesirable qualities, including low dynamic range, photoconversion of YFPs, photoactivation of CFPs during YFP excitation, phototoxicity during CFP excitation, and pH sensitivity^5^,^7^,^8^,^9^,^10^,^11^,^12^. Further, when imaging in cells, the wavelengths used to excite CFPs in CFP-YFP pairs induce significant autofluorescence from flavoproteins^13^. FRET pairs using green or yellow fluorescent proteins as donors and orange or red fluorescent proteins as acceptors (GFP-RFP pairs) are less commonly used, but exhibit fewer of the aforementioned disadvantages of CFP-YFP pairs^1^,^14^. Additionally, when performing FRET imaging in tissues, the GFP-RFP pair permits deeper imaging with less excitation power due to less light scattering of higher wavelength photons^15^,^16^. However, many GFP-RFP pairs have other limitations, including rapid photobleaching, and slow acceptor maturation^1^.

Previously, we developed a GFP-RFP pair, Clover and mRuby2, which offers FRET imaging with decreased phototoxicity and improved sensor responses over CFP-YFP pairs, and improved spectral properties over existing GFP-RFP pairs^1^. This GFP-RFP FRET pair has been used to image Zn^2+^ concentrations and CaMKIIα activity in living cells^2^,^17^. To date, Clover and mRuby2 remains the GFP-RFP pair with the highest FRET efficiency, FRET radius, and dynamic range in reporters^1^,^18^; however, this pair may be improved by increasing the photostability of Clover and the brightness of mRuby2. Developing new fluorophores with improved properties would benefit FRET imaging by increasing imaging time, signal-to-noise ratio, and dynamic range.

In this study, we aimed to optimize FRET sensing with GFP donors and RFP acceptors for mammalian expression. Starting with Clover and mRuby2, we engineered new proteins mClover3 and mRuby3, which feature improved properties for FRET imaging. We compared mRuby3 and mClover3 with other bright FPs for performance in mammalian cells, including newly described GFPs mNeonGreen, derived from *Branchiostoma lanceolatum*^18^ and Envy, derived from superfolder GFP and GFPgamma^19^. We show that mRuby3 is the brightest and most photostable monomeric RFP to date, and additionally offers high FRET efficiency in GFP-RFP fusions. Further, we find that mClover3 is a brighter and more photostable variant of Clover that compares favorably with other bright monomeric GFPs. Together, the mClover3-mRuby3 GFP-RFP pair improves FRET efficiency and improves dynamic responses in the calcium/calmodulin-dependent kinase II alpha (CaMKIIα) reporter, Camuiα^4^,^20^.

## Results

### Evolution of mRuby3

**Figure 1:**
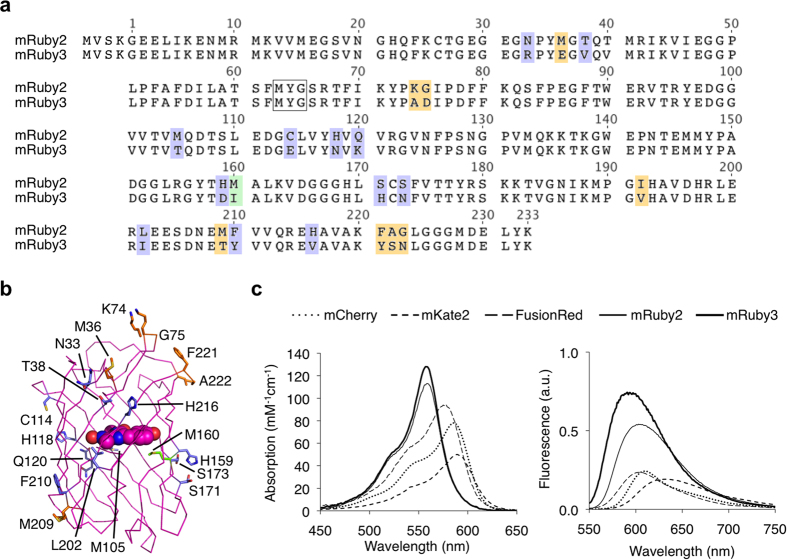
Development of mRuby3. **(a)** Sequence alignment of mRuby3 to its parent, mRuby2. The amino acids forming the chromophore are indicated by a black box. Outer barrel mutations are indicated in blue, inner barrel mutations are indicated in green, and loop mutations are indicated in orange. **(b)** Crystal structure of mRuby (PDB accession number 3U0M) showing mutations between mRuby2 and mRuby3. **(c)** Absorption (left) and emission (right) of mCherry, mKate2, FusionRed, mRuby2, and mRuby3. Absorbance spectra are scaled to peak extinction coefficient. Emission spectra are scaled so that areas under the curves are proportional to peak brightness (product of peak extinction coefficient and quantum yield).

mRuby2 is a favorable acceptor fluorophore in GFP-RFP FRET due to its high quantum yield, large Stokes shift, fast maturation, and high photostability^1^. We asked whether we could increase the brightness of mRuby2 in order to improve its performance as a FRET acceptor. We performed semi-rational evolution, performing mutagenesis in a combinatorial and simultaneous manner at several locations at once, sampling alleles found naturally in RFPs and choosing sites that were likely to impact protein folding or chromophore brightness based on their location in the structure. Specifically to improve brightness, we hypothesized that tight packing of residues near the chromophore could reduce excited-state vibrations that could lead to non-radiative decay. In each round, bacterial colonies were screened for high absorption and high fluorescence, and the best mutant was used as a template for the subsequent round. Across 6 rounds, we exhaustively screened sites with sequence variability among RFPs and with likely impact on fluorescent characteristics. The final best variant contains 21 substitutions relative to its parent mRuby2 (N33R, M36E, T38V, K74A, G75D, M105T, C114E, H118N, Q120K, H159D, M160I, S171H, S173N, I192V, L202I, M209T, F210Y, H216V, F221Y, A222S, G223N, Fig. 1a). Based on the crystal structure of mRuby, the majority of these mutations are located on the external barrel (Fig. 1b). This variant was monomeric, migrating similarly to the parental mRuby2 and FusionRed^21^ on gel filtration (Supplementary Fig. 1a). We designated this final variant mRuby3.

mRuby3 has excitation and emission maxima at 558 and 592 nm, respectively, which is blue-shifted compared to mRuby2 (Fig. 1c). The peak extinction coefficient is 128 mM^−1^ cm^−1^, and quantum yield is 0.45 (Table 1), resulting in a calculated brightness 35% greater than that of mRuby2 and establishing mRuby3 as the brightest monomeric RFP characterized to date. Notably, mRuby3 is highly photostable: under arc lamp illumination, mRuby3 had a half-life of 349 s (Supplementary Fig. 1b), compared to 123 s for mRuby2 and 337 s for TagRFP-T, previously the most photostable RFP. Like mRuby2, mRuby3 shows negligible reversibility and monoexponential photobleaching kinetics. Further, mRuby3 exhibits comparable acid tolerance compared to mRuby2, with a pKa of 4.8 (Supplementary Fig. 1c). Overall, these results indicate that mRuby3 is the brightest and most photostable monomeric RFP described to date.

### Performance of mRuby3 in mammalian cells

**Figure 2:**
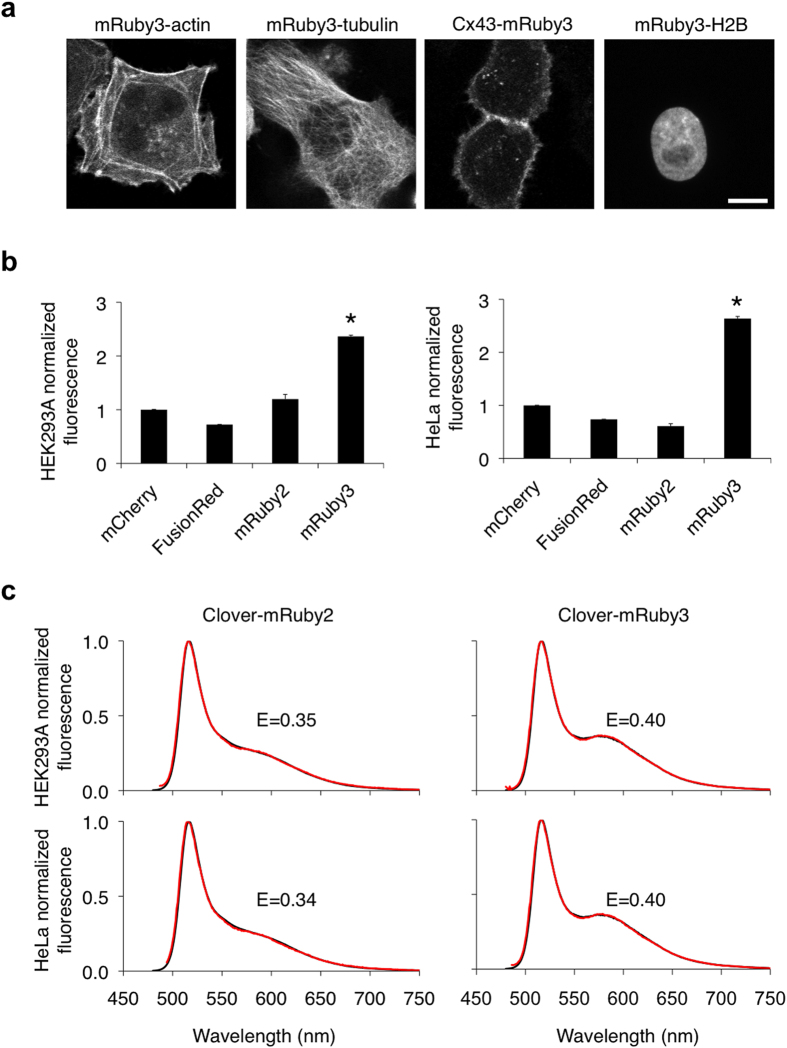
Performance of mRuby3 in mammalian cells. **(a)** Fluorescence images of HeLa cells expressing various mRuby3 fusion proteins that exhibit distinct subcellular localizations: mRuby3-7aa-actin (actin cytoskeleton), mRuby3-6aa-tubulin (microtubules), connexin43-7aa-mRuby3 (cell-cell adhesion junctions), mRuby3-10aa-H2B (nucleosomes). Linker lengths in amino acids are denoted by the number preceding ‘aa’. Scale bar, 10 μm. **(b)** Brightness comparison of monomeric RFPs in HEK293A and HeLa cells expressing mTurquoise2-P2A-RFP. The red fluorescence from each RFP, corrected by mTurquoise2, was normalized to that of mCherry. Data are presented as mean ± S.E.M. (n = 3). Asterisks indicate statistically significant differences (*p* < 0.05 by ANOVA followed by Dunnett’s post hoc tests). **(c)** FRET efficiency of Clover-mRuby2 compared to Clover-mRuby3 in HEK293A and HeLa cells. The emission spectra experimentally obtained (red lines) were fit to linear combinations of emission spectra of donor and acceptor (black lines).

Next, we systematically tested mRuby3 performance in mammalian cells. We first confirmed that a variety of mRuby3 fusions to critical subcellular targeting domains localized correctly in mammalian cells (Fig. 2a) and that fusions to histone H2B did not interfere with mitosis (Supplementary Fig. 2). We next compared fluorescence signals generated by mRuby3 to those of other monomeric RFPs in mammalian cells. While measurements of extinction coefficient and quantum yield on purified proteins *in vitro* allow an objective measure of per-molecule brightness of mature fluorescent protein, apparent brightness of fluorescent protein constructs in cells is also influenced by the efficiency of protein maturation and the half-life of the protein and thus should be empirically tested. We therefore compared the brightness of cells expressing various monomeric RFPs from a bicistronic construct allowing coexpression with mTurquoise2^22^, and used the mTurquoise2 signal to normalize for differences in mRNA levels. mRuby3 generated the highest signal when expressed in mammalian cells, outperforming mRuby2, FusionRed, and mCherry by over 100% in both HEK293A and HeLa cells (*p* < 0.05 by ANOVA followed by Dunnett’s post hoc tests; Fig. 2b). Interestingly, this degree of improvement was larger than expected from per-molecule brightness measurements alone. Our results thus suggest that, compared to other monomeric RFPs, mRuby3 is not only brighter per mature molecule, but also matures more completely or is more stable in mammalian cells.

Given these superior qualities, we theorized that mRuby3 would perform well as a FRET acceptor. mRuby2 has previously been shown to be a more effective FRET acceptor than several other monomeric RFPs, including mCherry^1^, mOrange^14^, and TagRFP^14^. Therefore, we were specifically interested in how mRuby3 performed compared to mRuby2. Using Clover as a FRET donor, the calculated Förster radius (r_0_) assuming random interfluorophore orientations was 6.5 nm for mRuby3 vs. 6.3 nm for mRuby2 (Table 2). To test whether mRuby3 was a more effective FRET acceptor for Clover in practice, we constructed fusion proteins consisting of Clover, a 15-aa flexible linker, and then mRuby2 or mRuby3. We then expressed each fusion protein in mammalian cells and derived FRET efficiency values by linear unmixing of emission spectra. FRET efficiency was higher with mRuby3 (E = 0.40) as the acceptor than with mRuby2 (E = 0.35) (Fig. 2c). These results show that mRuby3 produces higher brightness and functions as a more efficient FRET acceptor in mammalian cells than mRuby2.

### Evolution of mClover3

**Figure 3:**
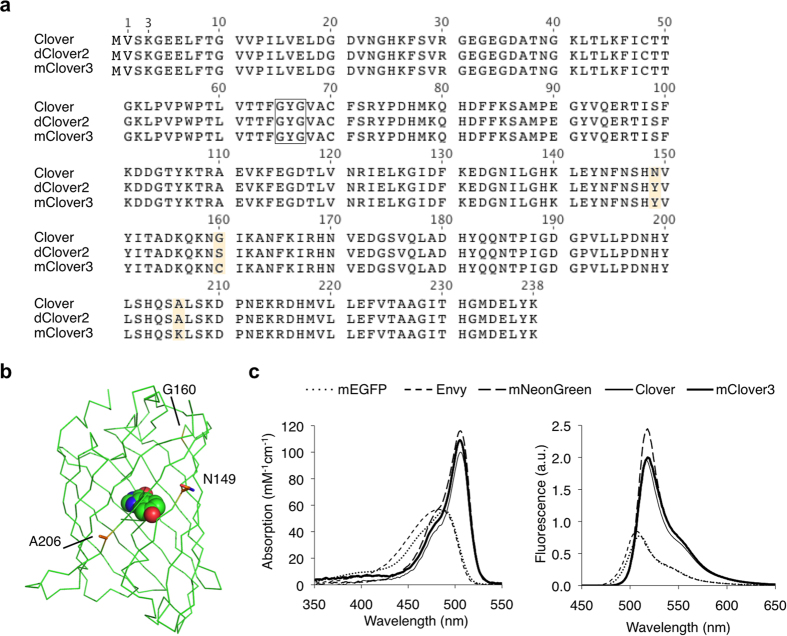
Development of mClover3. **(a)** Sequence alignment of mClover3 and dClover2 to its parent, Clover. The amino acids forming the chromophore are indicated by a black box. Outer barrel mutations are indicated in orange. **(b)** Crystal structure of superfolder GFP (PDB accession number 2B3P) showing mutations between Clover and mClover3. **(c)** Absorption (left) and emission (right) spectra of mEGFP, Envy, mNeonGreen, Clover, and mClover3. Absorbance spectra are scaled to peak extinction coefficient. Emission spectra are scaled so that areas under the curves are proportional to peak brightness (product of peak extinction coefficient and quantum yield).

We attempted to identify a GFP that would be superior to Clover in terms of brightness and photostability, and produce more efficient FRET to mRuby3. We hypothesized that a more photostable variant of Clover would function as an ideal donor for mRuby3, since the Clover-mRuby2 FRET pair had the highest r_0_ to date (Table 2). Screening random mutants for higher photostability on a home-made array of blue light-emitting diodes, we identified mutants N149Y (Clover1.5) and N149Y G160S (dClover2) with progressively higher photostability (Table 3). dClover2 had a brightness-normalized *in vitro* photostability half-life of 98 s compared to 50 s for Clover, as well as higher extinction coefficient (ε = 123 vs. 111 mM^–1^ cm^–1^) and quantum yield (φ = 0.80 vs. 0.76). However, while Clover was monomeric at 10 μM, Clover1.5 existed in a monomer-dimer equilibrium and dClover2 was dimeric (Supplementary Fig. 3a). Introduction of an A206K mutation, which had been effective in reducing the dimericity of other *Aequoria* GFP derivatives^23^, monomerized Clover1.5 but not dClover2 (Supplementary Fig. 3b). However, upon saturation mutagenesis at position 160 and screening for photostability and monomericity, we obtained an S160C variant that migrated as a monomer (Supplementary Fig. 3b). This variant, which we named mClover3 (Fig. 3a,b), exhibited a brightness-normalized *in vitro* bleaching half-life of 80 s (Supplementary Fig. 3c). Compared to the parental Clover, mClover3 had similar spectra (Fig. 3c), slightly higher brightness (ε = 109 vs. 111 mM^–1^ cm^–1^, φ = 0.78 versus 0.76, Table 1), and slightly reduced pH sensitivity (pK_a_ 6.5 vs 6.6, Supplementary Fig. 3d).

### Performance of mClover3 in mammalian cells

**Figure 4:**
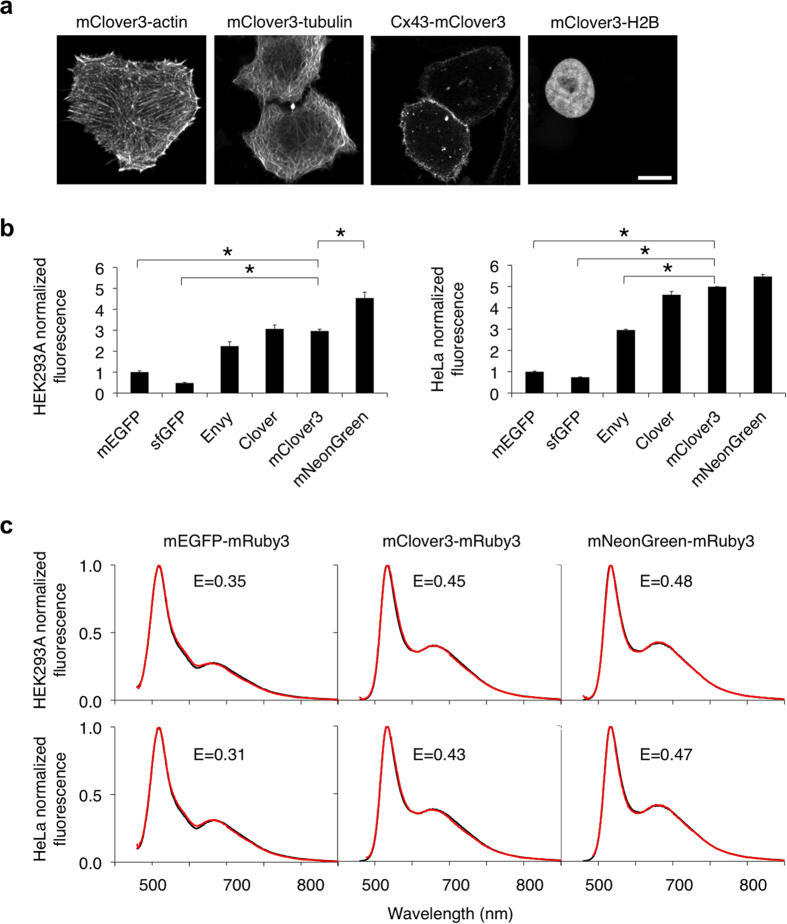
Performance of mClover3 in mammalian cells. **(a)** Fluorescence images of HeLa cells expressing various mClover3 fusion proteins that exhibit distinct subcellular localizations: mClover3-7aa-actin (actin cytoskeleton), mClover3-6aa-tubulin (microtubules), connexin43-7aa-mClover3 (cell-cell adhesion junctions), mClover3-10aa-H2B (nucleosomes). Linker lengths in amino acids are denoted by the number preceding ‘aa’. Scale bar, 10 μm. (**b**) Brightness comparison of GFPs in HEK293A and HeLa cells expressing GFP-P2A-mCherry. The green fluorescence from each GFP, corrected by mCherry fluorescence, was normalized to that of mEGFP. Data are presented as mean ± S.E.M. (n = 3). Asterisks indicate statistically significant differences (*p* < 0.05 by ANOVA followed by Dunnett’s post hoc tests). **(c**) FRET efficiencies of various GFP-mRuby3 tandem fusions in HEK293A and HeLa cells. The emission spectra experimentally obtained (red lines) were fit to linear combinations of emission spectra of donor and acceptor (black lines).

As with mRuby3, we systematically tested mClover3 performance in mammalian cells. First, we determined that a variety of mClover3 fusions to critical subcellular targeting domains localize correctly (Fig. 4a). Furthermore, mClover3 fusions to histone H2B did not perturb progression of mitosis (Supplementary Fig. 4a). Next, we compared Clover and mClover3 to other GFPs for brightness when expressed in mammalian cells. Among *Aequoria victoria* GFP variants, we chose mEGFP, superfolder GFP (sfGFP), and Envy for comparison. mEGFP is the most widely used fluorescent protein, sfGFP is an EGFP derivative with high folding efficiency^24^, and Envy was recently described as conferring higher brightness in budding yeast^19^. All of these have lower brightness than Clover or mClover3 in purified preparations *in vitro* (Table 1), but their apparent brightness in mammalian cells have not been compared. Among GFPs from other species, mNeonGreen, a recently described monomeric GFP derived from *B. lanceolatum* YFP, is 10% brighter than mClover3 *in vitro* (Table 1). mClover3 and mNeonGreen have very similar excitation and emission spectra which are slightly red-shifted relative to GFP, which could be due to the chromophores in both proteins engaging in cation-π interactions (with His-203 in Clover and Arg-197 in mNeonGreen)^18^.

Similarly to the previous comparison of mRuby3 with other RFPs, we compared the brightness of cells expressing various GFPs from a bicistronic construct allowing coexpression with mCherry, and used the mCherry signal to normalize for differences in mRNA levels. We observed that, in HEK293A cells, the highest mean fluorescence levels resulted from transfection by mNeonGreen, then Clover or mClover3, then Envy, then mEGFP, and finally sfGFP (Fig. 4b). The differences between mNeonGreen and mClover3, and between mClover3 and mEGFP or sfGFP, were statistically significant (*p* < 0.05 by ANOVA followed by Dunnett’s post hoc tests). In HeLa cells, expression of mNeonGreen, Clover, or mClover3 all resulted in similar levels of high fluorescence, followed by Envy, then mEGFP, and finally sfGFP (Fig. 4b). Here, the difference between mClover3 and Envy, mEGFP, or sfGFP was statistically significant (*p* < 0.05 by ANOVA followed by Dunnett’s post hoc tests). Thus, among GFPs, Clover, mClover3, and mNeonGreen produce the brightest fluorescence when expressed in mammalian cells.

We next determined which monomeric GFP would create the most optimal FRET donor to mRuby3. sfGFP was omitted due to its low fluorescence in mammalian cells, and Envy was not considered as it was revealed to be a dimer (Supplementary Fig. 4b). Calculated r_0_ values for mClover3-mRuby3 and mNeonGreen-mRuby3 pairs were both 6.5 nm, matching Clover-mRuby3, whereas the r_0_ value for mEGFP-mRuby3 was lower at 5.8 nm (Table 2). To determine which GFP is the best donor for mRuby3 in practice, we measured FRET efficiency in fusions with mRuby3 expressed in mammalian cells, comparing mClover3, mNeonGreen, and mEGFP. Consistent with the higher calculated r_0_ values, mClover3 and mNeonGreen resulted in higher FRET efficiencies than mEGFP in both HEK293A and HeLa cells (Fig. 4c). Interestingly, the FRET efficiencies of mClover3-mRuby3 and mNeonGreen-mRuby3 fusions were also higher than that of Clover-mRuby3 (Fig. 2c) despite the similar calculated r_0_ values, suggesting that mClover3 and mNeonGreen matured more completely than Clover when fused to mRuby3. Taken together, these results identify mClover3 and mNeonGreen as the most effective donors for mRuby3.

### Clover3-mRuby3 improves FRET sensing of CaMKIIα activity

**Figure 5:**
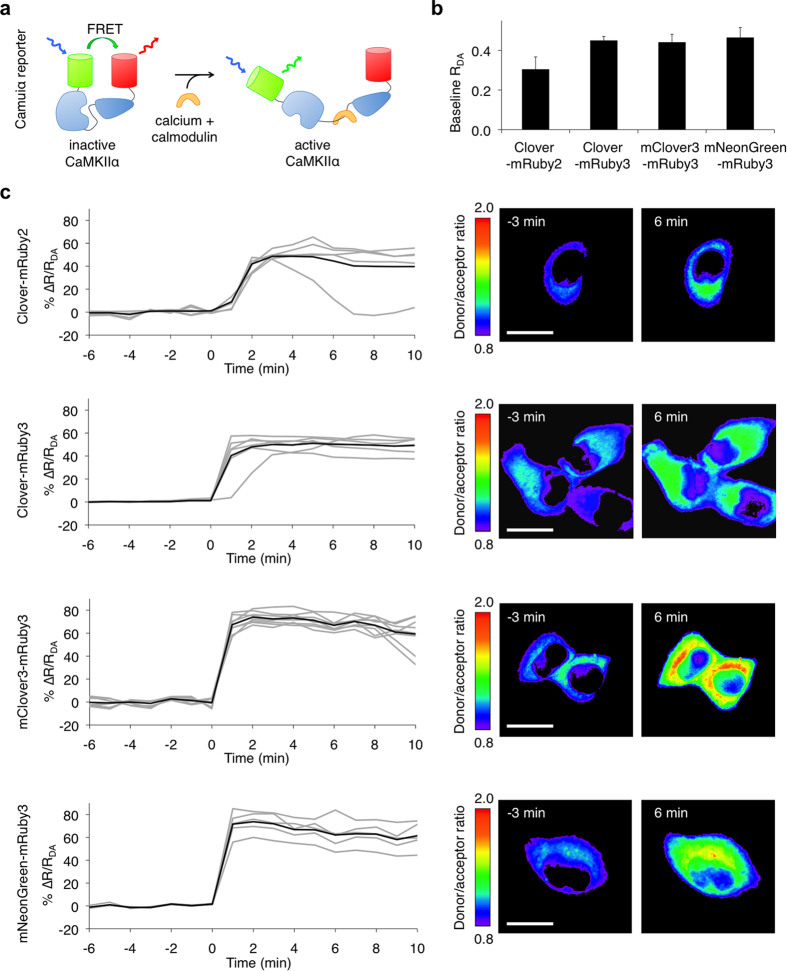
mClover3-mRuby3 and mNeonGreen3-mRuby3 improve responses in the CaMKIIα reporter Camuiα. (**a**) Organization of Camuiα with green/red FRET pairs. (**b**) Donor/acceptor emission ratio (R_DA_) for green/red Camuiα-expressed HeLa cells without ionomycin stimulation. Data are presented as mean ± S.E.M. (n = 11, 14, 17, 10 for Camuiα-CR, Camuiα-CR3, Camuiα-C3R3, and Camuiα-NR3 respectively). (**c**) Mean donor/acceptor emission ratio changes (ΔR/R_DA_) over time (left panel) and corresponding intensity-modulated ratiometric images (right panel) of HeLa cells stimulated with ionomycin. Emission ratio changes of individual cells are plotted as gray lines and the mean as black line. Data are presented as mean ± S.E.M. n = 7 cells for Camuiα-CR; n = 6 cells for Camuiα-CR3; n = 8 cells for Camuiα-C3R3; n = 5 cells for Camuiα-NR3. Difference in peak emission ratio change between Camuiα-CR and both Camuiα-C3R3 and Camuiα-NR3 is statistically significant (*p* < 0.05 by Student’s *t*-test). Scale bar, 20 μm.

The FRET reporter Camuiα has been useful for revealing the spatial distribution and time course of CaMKIIα activation in synaptic long-term potentiation and cardiomyocyte contraction^4^,^25^,^26^. In neurons and cardiomyocytes, researchers require the detection of FRET changes in small subcellular regions and in short time intervals, e.g. in 1-μm^2^ areas of synaptic spines and with 1-s sampling intervals. In such cases, photon collection and the response amplitude of the sensor are limiting factors in detecting activity changes. We thus sought to determine whether Camuiα performance could be improved with mRuby3 or mClover3. Camuiα is especially interesting as an example of a multimeric FRET reporter. Crystal structures reveal that CaMKIIα is a dodecameric doughnut-shaped protein with the C-terminal association domain of each chain facing toward the center and the N-terminal kinase domains arrayed at the edge, bound to a calmodulin-interacting regulatory segment and the association domain^27^. Upon calmodulin binding to the regulatory segment, the N-terminal kinase domains are released from interaction with the regulatory segment and increase their distance from the C-termini. In Camuiα, fluorescent proteins are fused to the termini of the CaMKIIα polypeptide to serve as a FRET pair. Consistent with the structure, Camuiα has high FRET in the inactive state and low FRET in the active state (Fig. 5a).

We hypothesized that using the new red and green FPs could improve Camuiα response, either by shifting the response to a more favorable part of the FRET efficiency curve or by improving maturation of the fluorophores. However, the multimeric structure of Camuiα could make its expression sensitive to interactions between fluorescent protein domains. Prior to this study, the variant of Camuiα with the largest response was Camuiα-CR, with Clover at the N-terminus and mRuby2 at the C-terminus^1^. We first tested whether Camuiα variants with new GFPs and mRuby3 could be well expressed. We created Camuiα-CR3 with Clover and mRuby3 as the FRET pair, Camuiα-C3R3 with mClover3 and mRuby3, and Camuiα-NR3 with mNeonGreen and mRuby3. All were well expressed with uniform cytoplasmic distribution (Supplementary Fig. 5a). In contrast, substitution of Clover with the more dimeric dClover2 caused aggregation (Supplementary Fig. 5a). Interestingly, dClover2 did not interfere with mitosis when fused to histone H2B (Supplementary Fig. 5b), indicating that the Camuiα reporter is especially sensitive to fluorescent protein dimerization.

We next tested the ability of the new red and green FPs to improve baseline FRET in the Camuiα reporter. Camuiα-CR3 with mRuby3 exhibited higher basal FRET than Camuiα-CR with mRuby2 (Fig. 5b). This was expected for two reasons. First, the larger r_0_ of Clover-mRuby3 compared to Clover-mRuby2 (6.5 nm vs. 6.3 nm) would be expected to increase basal FRET. Second, mRuby3 matured significantly better than mRuby2 in multiple contexts (Fig. 2b), and more complete maturation within Camuiα would also be expected to increase basal FRET. Substitution of mClover3 or mNeonGreen for Clover did not additionally alter basal FRET levels significantly (Fig. 5b), consistent with the negligible differences in r_0_ among Clover-mRuby3, mClover3-mRuby3, and mNeonGreen-mRuby3 pairs, and implying no major differences in maturation efficiency of these GFPs within Camuiα.

Finally, to determine if the performance of Camuiα could be improved with the new FRET pairs, we compared responses of Camuiα-CR, -CR3, -C3R3, and -NR3 in HeLa cells stimulated with ionomycin. The average response of Camuiα-CR3 (measured as the change in the donor/acceptor emission ratio between inactive and active states) was similar to that of Camuiα-CR, at approximately 45% (Fig. 5c). As FRET was higher in the inactive state (Fig. 5b) in Camuiα-CR3, this result implies that FRET in the active state was also higher in Camuiα-CR3. In contrast, using mClover3 or mNeonGreen in place of Clover significantly improved Camuiα dynamic range, enhancing the calcium-induced increase in the green/red emission ratio from 45% in Camuiα-CR3 to 70% in Camuiα-C3R3 and Camuiα-NR3, a 56% increase (*p* < 0.05 by Student’s *t*-test; Fig. 5c). This improvement in Camuiα responsiveness was unexpected given the similar r_0_ values and similar baseline FRET efficiencies of Camuiα-CR3, -C3R3, and -NR3, so we speculate that the inactive state of Camuiα could be differentially stabilized by different fluorescent proteins at the N-terminus. For example, weak interaction between closely juxtaposed surfaces of Clover monomers in adjacent subunits could stabilize Camuiα-CR in its closed high-FRET conformation, disfavoring transition into the open conformation. This effect may be absent or reduced in the unrelated mNeonGreen and in mClover3 due to the N149Y and A206K surface mutations, facilitating the transition to the open active conformation in Camuiα-C3R3 and Camuiα-NR3.

## Discussion

In this study, we have created new red and green fluorescent proteins with superior performance characteristics for cell or protein labeling and for FRET. Starting from mRuby2, previously the brightest red fluorescent protein, we have engineered mRuby3, with higher brightness, higher photostability, and higher expression in mammalian cells. To our knowledge, mRuby3 is the brightest and most photostable monomeric RFP yet described. Starting from Clover, the brightest derivative of jellyfish GFP, we have engineered a more photostable derivative, mClover3.

Engineering bright FPs with high photostability has been challenging due to the lack of rational methods for predicting the effects of specific mutations on photostability, although limiting oxygen access to the FP’s chromophore has been proposed as a mechanism for improving photostability^11^. In mRuby3, the M160I mutation replaces a linear aliphatic side chain beneath the chromophore (when the β-barrel is oriented with termini pointing upwards) with a branched one. This could result in more rigid packing of the protein around the chromophore, which in turn could produce higher brightness by suppressing chromophore motions and higher photostability by reducing room for oxygen near the chromophore. In mClover3, the external-facing Tyr side chain at amino acid 149 on β-strand 7 might hinder oxygen access to the chromophore, as the gap between β-strand 7 and 8 has been identified as a path for oxygen entry into the β-barrel and chromophore bleaching^28^,^29^. In contrast, it is not easy to rationalize the ability of position 160 (homologous to position 153 in mRuby3) in Clover-family GFPs to influence photostability (mClover3 is more photostable than mClover1.5, and the two differ only at this position). Position 160 is located 1.5 nm from the chromophore at the beginning of β-strand 8. One possibility is that Cys-160 exerts long-distance effects on the conformation of β-strand 7 or 8 near the chromophore to restrict oxygen access. Another is that Cys-160 blocks a previously uncharacterized pathway for oxygen entry through the end of the β-barrel. Future studies aimed at solving the crystal structures of mClover3 and mRuby3 and performing molecular dynamics simulations of oxygen diffusion may be helpful to understand how these mutations affect brightness and photostability.

The similarities between mClover3 and mNeonGreen, two GFPs derived from different natural parents, are striking. mClover3 is derived from *A. victoria* GFP (avGFP) while mNeonGreen is derived from a lancelet YFP. The natural parental proteins were different in wavelength tuning (ex/em peaks of 395/509 nm and 513/524 nm for GFP and LanYFP, respectively)^18^,^30^, and showed low sequence conservation (14% identity). Yet mClover3 and mNeonGreen are similar in brightness and spectra and are the two brightest monomeric FPs characterized. This suggests that they are likely to represent a local peak in the FP fitness landscape for brightness. mTurquoise2 CFP has a higher QY (0.93), likely because the CFP chromophore may benefit in terms of QY by being composed of two fused rings, whose orientation can be anchored by the hydrogen bond introduced in mTurquoise2. In GFPs, a primary source of non-radiative decay is rotation of the phenolate group relative to the imidazolinone group around the methylene bridge. The phenolate group in GFP lacks chemical groups in the ortho and para positions that can participate in hydrogen bonding that could restrain rotational motions. The observed QYs of GFP-type chromophores are all 0.70–0.80 in mClover3, mNeonGreen, and YFPs, suggesting these proteins have reached a quantum yield limit imposed by the torsional flexibility of the chromophore. However, compared to CFPs, mClover3, mNeonGreen, and YFPs have higher peak extinction coefficients, allowing their overall peak brightness values to surpass those of CFPs.

mClover3 or mNeonGreen paired with mRuby3 have several advantages over previous pairs for FRET. First of all, they have the highest calculated r_0_ of any ratiometric FRET pairs yet described, and a high r_0_ has been shown to improve responses of FRET reporters that operate in a range of low FRET efficiency^1^. Second, mRuby3 demonstrates more complete maturation in mammalian cells than mRuby2, as evidenced by an increase in fluorescence of cells expressing mRuby3 that is more than proportional to its increase in molar brightness. This increased maturation would be expected to increase FRET efficiency as well. Finally, as mClover3 and mNeonGreen are more photostable than Clover, and as mRuby3 is more photostable than mRuby2, mClover3 or mNeonGreen paired with mRuby3 should be generally advantageous for time-lapse FRET imaging as well.

The usefulness of mRuby3 and mClover3 or mNeonGreen should extend to the multitude of applications of green and red fluorescent proteins in general. With its very high photostability and large Stokes shift, mRuby3 should be an excellent probe for time-lapse imaging and detection of proteins in photon-limited situations, including fast time-lapse and single-molecule imaging. With its improved maturation, mRuby3 may also be more amenable than mRuby2 to circular permutation, facilitating the engineering of biosensors based on circularly permuted RFPs such as R-CaMP, which was based on mRuby^31^.

In addition, mClover3 or mNeonGreen should be a useful substitute for EGFP to improve the brightness of fusion proteins. mNeonGreen exhibits slightly higher (6%) molar brightness than mClover3, and therefore remains the brightest fluorescent protein characterized. In addition, mNeonGreen is more photostable than mClover3, and thus may be preferable when high-intensity time-lapse imaging is performed. On the other hand, mClover3 could be useful for improving the brightness of sensors based on circularly permuted jellyfish GFP, from which mClover3 was derived. Jellyfish GFP and its derivatives have proven to be compatible with circular permutation in β-strand 7 near the chromophore, which allows for conformational changes in linked domains to influence chromophore absorbance, such as in the calcium sensor G-CaMP^32^ or the membrane voltage sensor ASAP^33^. In contrast, circular permutation at similar sites in monomeric GFPs engineered from naturally multimeric precursors, such as mAG, Dronpa, or mNeonGreen, has yet to be reported.

In summary, the new proteins reported here, mRuby3 and mClover3, should be useful red and green fluorescent proteins whenever detection sensitivity, photostability, or folding robustness is desired. In particular, mRuby3 is the brightest and most photostable monomeric RFP yet characterized, and functions well alone, within fusion proteins, and within FRET sensors, and should be the preferred RFP in a broad range of applications.

## Methods

### FRET-distance modeling

To model FRET-distance relationships, we calculated theoretical emission spectra using the equation *f*_total_(λ) = ε_D_(λ_ex_)[(1 − *E*) × φ_D_ × *f*_D_(λ) + *E* × φ_A_ × *f*_A_(λ)] + ε_A_(λ_ex_) × *f*_A_(λ), where *f*_total_ is fluorescence at wavelength λ, ε_D_(λ_ex_) is extinction coefficient of the donor at the excitation wavelength, *E* is FRET efficiency, φ_D_ is quantum yield of the donor, *f*_D_(λ) is normalized emission of the donor at wavelength λ, φ_A_ is quantum yield of the acceptor, *f*_A_(λ) is normalized emission of the acceptor at wavelength λ and ε_A_(λ_ex_) is extinction coefficient of the acceptor at the excitation wavelength. *E* for a measured emission spectrum was then determined as the value of *E* (to two significant digits) that gave the best fit between the theoretical and measured emission spectra. An apparent interfluorophore distance r was then calculated from the Förster equation *E* = 1/(1 + (*r*^6^ /*r*_0_^6^)), where r_0_ is the Föster radius.

### Plasmid construction

Plasmids were constructed by standard molecular biology methods, including PCR, overlap extension PCR, restriction fragment ligation, and In-Fusion cloning (Clontech). All cloning junctions and PCR products were sequence verified. Further details are described in the methods descriptions for specific constructs below. Complete plasmid sequences are available upon request, and plasmids will be distributed through Addgene (addgene.org).

### HeLa and HEK293A cell culture and transfection

Cells were maintained in high-glucose Dulbecco’s Modified Eagle Medium (DMEM, HyClone) supplemented with 5–10% fetal bovine serum (Gemini), 2 mM glutamine (Gemini), 100 U/mL penicillin and 100 μg/mL streptomycin (Gemini) at 37 °C with 5% CO_2_. Cells were transfected at 60-80% confluence with Lipofectamine 2000 (Life Tech) in Opti-MEM (Gibco). For Figs 2b, 4b and 5, cells were transfected in 6- or 12-well plates, and the medium was refreshed 3–5 hours after transfection, unless otherwise noted. 24 hours later, cells were split into Lab-Tek 8-chamber coverglass and cultured for another 24 hours before experiments were performed. For Figs 2a and 4a and Supplementary Figs 2 and 4, cells were either transfected in 35 mm 4-chamber #1 coverglass dishes (*In Vitro* Scientific), or transfected in 12-well plates and subsequently split into 4-chamber dishes.

### Fluorescence microscopy of fusion proteins

For mClover3 and mRuby3 fusions, HeLa cells were imaged 24-72 hours post-transfection, in FluoroBrite DMEM (Gibco) supplemented with B-27 (Gibco), in a humidified chamber maintained at 33 °C. Micrographs were acquired on an FV1000 confocal microscope, using a UPLFNL 40× oil-immersion NA 1.3 objective (Olympus). Scan resolution was 2048 × 2048 pixels, with speed 2 μs/pixel, and Kalman filtering of 2–4 iterations. Pinhole diameter was adjusted dynamically by Olympus software. For mClover3, 488 nm laser excitation was used, with 500-600 nm light collected. For mRuby3, 559 nm laser excitation was used, with 570-670 nm light collected. Z-stacks of step size 0.5–1.0 μm were acquired. Some micrograph panels derive from maximum intensity projections of multiple z-slices. Image processing was performed with ImageJ. For dClover2 fusions, HeLa (S3 line) cells were grown in Dulbecco’s modified Eagle’s medium (DMEM, Invitrogen) supplemented with 12.5% FBS (HyClone). Transfections were performed using Effectene (Qiagen) following the manufacturer’s protocol. Cells were maintained under a humidified atmosphere of 5% CO_2_ in air in Delta-T culture chambers (Bioptechs) during imaging. Single-image microscopy of fusion proteins was performed using a Nikon 80i microscope (widefield) equipped with a Chroma FITC filter set.

### *In vitro* protein characterization

Fluorescent proteins with hexahistidine N-terminal tags were purified with HisPur Cobalt Resin (Pierce) and desalted into PBS (pH = 7.2) using Econo-Pac 10DG desalting columns (Bio-Rad). Absorbance, excitation and emission spectra were measured with Tecan plate readers (Safire2 or Infinite M1000 Pro). Extinction coefficients were calculated using previously described base-denaturation method^34^. Quantum yields were determined using Clover (for all Clover variants) and mRuby2 (for mRuby3) as standards. pH titrations were performed using a series of buffers (1 M HOAc, 1 M NaOAc, 5 M NaCl for pH 3.0-4.5; 1 M NaH2PO4, 1 M Na2HPO4, 5 M NaCl for pH 5-9.0; 100 mM glycine for pH 9.5 and 10).

*In vitro* photobleaching measurements were performed on purified proteins in PBS in mineral oil droplets using an Olympus IX81 inverted microscope with a 40 × 0.90-NA UPlan S-Apo objective, an X-Cite 120-W metal halide lamp (Lumen Dynamics) at 100% neutral density passed through a 545/30 nm excitation filter (Chroma) for mRuby variants and a 485/30 nm excitation filter (Omega) for Clover variants. Images were acquired every 1 s under continuous illumination using a cooled ORCA-ER CCD (Hamamatsu). Times were adjusted to produce photon output rates of 1,000 per molecule per s as previously described^35^.

Gel filtration chromatography was performed with a Superdex 200 30/100 GL column (GE health). 100 μL of 10 μM purified proteins were loaded and eluted at a flow rate of 0.5 ml/min. Protein elution was monitored by absorbance at 280 nm.

### Basal FRET measurements

GFP-RFP tandem fusions consisted of C-terminus truncated avGFP derivatives (no ‘GITHGMDELYK’) or mNeonGreen (no ‘GMDELYK’) fused to aa 3-233 of mRuby2 or mRuby3 via the linker sequence LESGGEDPMVSKGEE. Tandem fusions were expressed in HEK293A and HeLa cells using Lipofectamine 2000 (Life Technologies). At 2 days post-transfection, cells were transferred to a transparent-bottom 96-well plate and fluorescence spectra were obtained on an Infinite M1000 PRO microplate reader (Tecan). Emission spectra was obtained between 490 and 750 nm using 470 nm excitation light. A 5-nm band pass was used for both excitation and emission.

### Brightness comparison of mutants in mammalian cells

Comparisons of green FPs and mRuby variants brightness in mammalian cells were made using mCherry and mTurquoise2 as an expression level reference, respectively. Fusions were expressed in HEK293A and HeLa cells using Lipofectamine 2000 (Life Technologies). At 2 days post-transfection, cells were transferred to a transparent-bottom 96-well plate and fluorescence spectra or intensity under a given wavelength were obtained on an Infinite M1000 PRO microplate reader (Tecan). The ex and em monochromators settings for different FPs are as follows (FP-ex-em): mTurquoise2-434/5 nm-474/5 nm, mCherry-587/20 nm-610/5 nm, GFP-430/20 nm-480~650 nm, RFP-550/10 nm-570~670 nm. Relative brightness was calculated from integrated green FPs or mRuby variants emission divided by peak mCherry or mTurquoise2 emission.

### Camuiα sensor improvement and characterization

To construct Camuiα-CR variants, C-terminus truncated avGFP derivatives (no ‘GITHGMDELYK’) or mNeonGreen (no ‘GMDELYK’) were amplified with an N-terminal NheI site and a C-terminal extension encoding the linker between the GFP and the CaMKIIα domain. The CaMKIIα domain flanked by linkers on either end was PCR amplified. mRuby2 or mRuby3 (aa 1-237) was PCR amplified with an N-terminal extension encoding the linker following the CaMKIIα domain and with an ApaI restriction site at the C terminus. The full insert was assembled by overlap PCR and cloned into the pcDNA3.1 backbone using restriction sites NheI and ApaI.

For microscopy experiments, cells were cultured and transfected in chamber slides and imaged 2 d following transfection. Cells were serum starved in serum-free DMEM for 4 h and then washed two times with Hank’s Balanced Salt Solution (HBSS, HyClone) and maintained in HBSS with 2 mM calcium. Cells were imaged using a cooled ORCA-ER CCD camera (Hamamatsu) and a 40 × 1.2-NA C-Apochromat water-immersion objective on an Axiovert 200 M inverted microscope (Zeiss) controlled by Micro-manager 1.4 software^36^ on a 17-inch 2.5-GHz Core 2 Duo MacBook Pro running Mac OS 10.6.8 (Apple). Illumination was provided by an Exfo metal-halide light source. Consecutive FRET and donor emission images were acquired with the following filters (ex, excitation; em, emission): ex HQ470/30 nm (Chroma) and em 505AELP nm (Omega) for GFP, and HQ470/30 nm (Chroma) and em BA575IF nm (Olympus) for FRET. After baseline acquisition for 6 min, cells were stimulated with 1 μM of the calcium ionophore ionomycin, and images were acquired for 10 min.

### Ratiometric image analysis

FRET measurements were quantified using ImageJ (NIH). Raw 16-bit TIFF files were imported into ImageJ, then regions were drawn on random transfected cells for reporter responses and on nontransfected cells for background measurements. Emission ratios were obtained by calculating background-subtracted donor intensities divided by background-subtracted FRET intensities. Time-course ratio measurements were normalized to baseline prestimulation values. Intensity-modulated displays were generated using a full-spectrum lookup table with minimum values in blue and maximum values in red and with intensity modulation by the acceptor channel.

### Statistical methods

ANOVA followed by Dunnett’s post hoc tests were used to determine the differences between brightness measurements in mammalian cells. Student’s *t*-test was used to determine the differences between peak emission ratio changes of Camuiα variants. Statistical analysis was performed in Excel (Microsoft) and Prism (GraphPad).

## Additional Information

**How to cite this article**: Bajar, B. T. *et al.* Improving brightness and photostability of green and red fluorescent proteins for live cell imaging and FRET reporting. *Sci. Rep.* **6**, 20889; doi: 10.1038/srep20889 (2016).

## Supplementary Material

Supplementary Information

## Figures and Tables

**Table 1:** Photophysical properties of bright and monomeric green and red fluorescent proteins.

Protein	Ref.^a^	λ_ex_^b^	λ_em_^c^	ε^d^	φ^e^	Brightness^f^	pK_a_^g^	Photostability^h^
Envy	19	485^i^	508^i^	57^i^	0.65^i^	37^i^	ND	113^i^
mEGFP	37	488	507	56	0.60	34	6.0	150
sfGFP	24	485	510	83	0.65	54	ND	ND
Clover	1	505	515	111	0.76	84	6.6	50
**mClover3**	**This study**	**506**	**518**	**109**	**0.78**	**85**	**6.5**	**80**
mNeonGreen	18	506	517	116	0.80	90	5.7	158
TagRFP-T	11	555	584	81	0.41	33	4.6	337
mRuby2	1	559	600	113	0.38	43	4.7	123
**mRuby3**	**This study**	**558**	**592**	**128**	**0.45**	**58**	**4.8**	**349**
FusionRed	21	580	608	94.5	0.19	18	4.6	176
mCherry	37	587	610	72	0.22	16	<4.5	96
mKate2	37	588	633	63	0.40	25	5.4	84

^a^Source of data unless otherwise noted.

^b^Excitation maximum in nm.

^c^Emission maximum in nm.

^d^Peak extinction coefficient in mM^−1^ cm^−1^ determined by alkali-denaturation.

^e^Fluorescence quantum yield.

^f^Product of ε and φ.

^g^pH at which fluorescence intensity is 50% of its maximum value.

^h^Time in s to photobleach from 1,000 to 500 photons per s per molecule under arc-lamp illumination.

^i^Data from this study. ND, not determined.

**Table 2:** r_0_ values for GFP-RFP FRET pairs.

FRET pair	φ_D_^a^	ε_A_^b^	r_0_^c^ (nm)
mEGFP-mCherry	0.60	72	5.4^d^
Clover-mCherry	0.76	72	5.8^d^
Clover-mRuby2	0.76	113	6.3^d^
mEGFP-mRuby3	0.60	128	5.8
Clover-mRuby3	0.76	128	6.5
mClover3-mRuby3	0.78	128	6.5
mNeonGreen-mRuby3	0.80	128	6.5

^a^Quantum yield of donor.

^b^Extinction coefficient of acceptor in mM^−1^ cm^−1^.

^c^Calculated Förster radius assuming random interfluorophore orientation (κ^2^ = 2/3).

^d^Data from ref. 1.

**Table 3:** Evolution of mClover3.

Clover variant	ε^a^	φ^b^	Brightness^c^	Stoichiometry^d^	Photostability^e^
Clover	111	0.76	84	monomer	50
Clover N149Y (Clover1.5)	118	0.78	92	monomer-dimer	66
Clover1.5 A206K (mClover1.5)	109	0.79	86	monomer	66
Clover1.5 G160S (dClover2)	123	0.80	98	dimer	98
dClover2 A206K	117	0.78	91	dimer	95
dClover2 A206K S160C (mClover3)	109	0.78	85	monomer	80

^a^Extinction coefficient at peak in units of mM^−1^ cm^−1^.

^b^Total quantum yield.

^c^Product of ε and φ.

^d^By size-exclusion chromatography at a loading concentration of 10 μM.

^e^Time in s to photobleach from 1,000 to 500 photons per s per molecule under arc-lamp illumination.

## References

[b1] Lam A. J. *et al.* Improving FRET dynamic range with bright green and red fluorescent proteins. Nat Methods 9, 1005–1012 (2012). 22961245 10.1038/nmeth.2171 PMC3461113

[b2] Miranda J. G. *et al.* New alternately colored FRET sensors for simultaneous monitoring of Zn^2+^ in multiple cellular locations. PLoS One 7, e49371 (2012). 23173058 10.1371/journal.pone.0049371 PMC3500285

[b3] Ni Q., Titov D. V. & Zhang J. Analyzing protein kinase dynamics in living cells with FRET reporters. Methods 40, 279–286 (2006). 16908183 10.1016/j.ymeth.2006.06.013

[b4] Takao K. *et al.* Visualization of synaptic Ca2+/calmodulin-dependent protein kinase II activity in living neurons. J Neurosci 25, 3107–3112 (2005). 15788767 10.1523/JNEUROSCI.0085-05.2005 PMC6725094

[b5] Tsutsui H., Karasawa S., Okamura Y. & Miyawaki A. Improving membrane voltage measurements using FRET with new fluorescent proteins. Nat Methods 5, 683–685 (2008). 18622396 10.1038/nmeth.1235

[b6] Zhang J., Ma Y., Taylor S. S. & Tsien R. Y. Genetically encoded reporters of protein kinase A activity reveal impact of substrate tethering. Proc Natl Acad Sci USA 98, 14997–15002 (2001). 11752448 10.1073/pnas.211566798 PMC64972

[b7] Dixit R. & Cyr R. Cell damage and reactive oxygen species production induced by fluorescence microscopy: effect on mitosis and guidelines for non-invasive fluorescence microscopy. Plant J 36, 280–290 (2003). 14535891 10.1046/j.1365-313x.2003.01868.x

[b8] Malkani N. & Schmid J. A. Some secrets of fluorescent proteins: distinct bleaching in various mounting fluids and photoactivation of cyan fluorescent proteins at YFP-excitation. PLoS One 6, e18586 (2011). 21490932 10.1371/journal.pone.0018586 PMC3072413

[b9] Raarup M. K. *et al.* Enhanced yellow fluorescent protein photoconversion to a cyan fluorescent protein-like species is sensitive to thermal and diffusion conditions. J Biomed Opt 14, 034039 (2009). 19566331 10.1117/1.3103338

[b10] Reiff D. F. *et al.* *In vivo* performance of genetically encoded indicators of neural activity in flies. J Neurosci 25, 4766–4778 (2005). 15888652 10.1523/JNEUROSCI.4900-04.2005 PMC1464576

[b11] Shaner N. C. *et al.* Improving the photostability of bright monomeric orange and red fluorescent proteins. Nat Methods 5, 545–551 (2008). 18454154 10.1038/nmeth.1209 PMC2853173

[b12] Sinnecker D., Voigt P., Hellwig N. & Schaefer M. Reversible photobleaching of enhanced green fluorescent proteins. Biochemistry 44, 7085–7094 (2005). 15865453 10.1021/bi047881x

[b13] Hockberger P. E. *et al.* Activation of flavin-containing oxidases underlies light-induced production of H2O2 in mammalian cells. Proc Natl Acad Sci USA 96, 6255–6260 (1999). 10339574 10.1073/pnas.96.11.6255 PMC26868

[b14] George Abraham B. *et al.* Fluorescent Protein Based FRET Pairs with Improved Dynamic Range for Fluorescence Lifetime Measurements. PLoS One 10, e0134436 (2015). 26237400 10.1371/journal.pone.0134436 PMC4523203

[b15] Helmchen F. & Denk W. Deep tissue two-photon microscopy. Nat Methods 2, 932–940 (2005). 16299478 10.1038/nmeth818

[b16] Rose T., Goltstein P. M., Portugues R. & Griesbeck O. Putting a finishing touch on GECIs. Front Mol Neurosci 7, 88 (2014). 25477779 10.3389/fnmol.2014.00088 PMC4235368

[b17] Yagishita S. *et al.* A critical time window for dopamine actions on the structural plasticity of dendritic spines. Science 345, 1616–1620 (2014). 25258080 10.1126/science.1255514 PMC4225776

[b18] Shaner N. C. *et al.* A bright monomeric green fluorescent protein derived from Branchiostoma lanceolatum. Nat Methods 10, 407–409 (2013). 23524392 10.1038/nmeth.2413 PMC3811051

[b19] Slubowski C. J., Funk A. D., Roesner J. M., Paulissen S. M. & Huang L. S. Plasmids for C-terminal tagging in Saccharomyces cerevisiae that contain improved GFP proteins, Envy and Ivy. Yeast 32, 379–387 (2015). 25612242 10.1002/yea.3065 PMC4390471

[b20] Kwok S. *et al.* Genetically encoded probe for fluorescence lifetime imaging of CaMKII activity. Biochem Biophys Res Commun 369, 519–525 (2008). 18302935 10.1016/j.bbrc.2008.02.070 PMC2396457

[b21] Shemiakina I. I. *et al.* A monomeric red fluorescent protein with low cytotoxicity. Nat Commun 3, 1204 (2012). 23149748 10.1038/ncomms2208

[b22] Goedhart J. *et al.* Structure-guided evolution of cyan fluorescent proteins towards a quantum yield of 93%. Nat Commun 3, 751 (2012). 22434194 10.1038/ncomms1738 PMC3316892

[b23] Zhang J., Campbell R. E., Ting A. Y. & Tsien R. Y. Creating new fluorescent probes for cell biology. Nat Rev Mol Cell Biol 3, 906–918 (2002). 12461557 10.1038/nrm976

[b24] Pedelacq J. D., Cabantous S., Tran T., Terwilliger T. C. & Waldo G. S. Engineering and characterization of a superfolder green fluorescent protein. Nat Biotechnol 24, 79–88 (2006). 16369541 10.1038/nbt1172

[b25] Erickson J. R., Patel R., Ferguson A., Bossuyt J. & Bers D. M. Fluorescence resonance energy transfer-based sensor Camui provides new insight into mechanisms of calcium/calmodulin-dependent protein kinase II activation in intact cardiomyocytes. Circ Res 109, 729–738 (2011). 21835909 10.1161/CIRCRESAHA.111.247148 PMC3182829

[b26] Lee S. J., Escobedo-Lozoya Y., Szatmari E. M. & Yasuda R. Activation of CaMKII in single dendritic spines during long-term potentiation. Nature 458, 299–304 (2009). 19295602 10.1038/nature07842 PMC2719773

[b27] Lisman J., Schulman H. & Cline H. The molecular basis of CaMKII function in synaptic and behavioural memory. Nat Rev Neurosci 3, 175–190 (2002). 11994750 10.1038/nrn753

[b28] Chapagain P. P., Regmi C. K. & Castillo W. Fluorescent protein barrel fluctuations and oxygen diffusion pathways in mCherry. J Chem Phys 135, 235101 (2011). 22191901 10.1063/1.3660197 PMC3248888

[b29] Regmi C. K., Bhandari Y. R., Gerstman B. S. & Chapagain P. P. Exploring the diffusion of molecular oxygen in the red fluorescent protein mCherry using explicit oxygen molecular dynamics simulations. J Phys Chem B 117, 2247–2253 (2013). 23363049 10.1021/jp308366y PMC3587716

[b30] Tsien R. Y. The green fluorescent protein. Annu Rev Biochem 67, 509–544 (1998). 9759496 10.1146/annurev.biochem.67.1.509

[b31] Akerboom J. *et al.* Genetically encoded calcium indicators for multi-color neural activity imaging and combination with optogenetics. Front Mol Neurosci 6, 2 (2013). 23459413 10.3389/fnmol.2013.00002 PMC3586699

[b32] Nakai J., Ohkura M. & Imoto K. A high signal-to-noise Ca(2+) probe composed of a single green fluorescent protein. Nat Biotechnol 19, 137–141 (2001). 11175727 10.1038/84397

[b33] St-Pierre F. *et al.* High-fidelity optical reporting of neuronal electrical activity with an ultrafast fluorescent voltage sensor. Nat Neurosci 17, 884–889 (2014). 24755780 10.1038/nn.3709 PMC4494739

[b34] Gross L. A., Baird G. S., Hoffman R. C., Baldridge K. K. & Tsien R. Y. The structure of the chromophore within DsRed, a red fluorescent protein from coral. Proc Natl Acad Sci USA 97, 11990–11995 (2000). 11050230 10.1073/pnas.97.22.11990 PMC17282

[b35] Shaner N. C., Steinbach P. A. & Tsien R. Y. A guide to choosing fluorescent proteins. Nat Methods 2, 905–909 (2005). 16299475 10.1038/nmeth819

[b36] Edelstein A., Amodaj N., Hoover K., Vale R. & Stuurman N. Computer control of microscopes using microManager. Curr Protoc Mol Biol Chapter 14, Unit14.20 (2010). 10.1002/0471142727.mb1420s92 PMC3065365 20890901

[b37] Day R. N. & Davidson M. W. The fluorescent protein palette: tools for cellular imaging. Chem Soc Rev 38, 2887–2921 (2009). 19771335 10.1039/b901966a PMC2910338

