# Supplementary material for: Improving brightness and photostability of green and red fluorescent proteins for live cell imaging and FRET reporting
Source: Sci Rep. 2016 Feb 16;6:20889. doi: 10.1038/srep20889 (PMC4754705; doi:10.1038/srep20889)
Supplement: Supplementary Information [file srep20889-s1.docx]

**Improving brightness and photostability of green and red fluorescent proteins for live cell imaging and FRET reporting**

Bryce T. Bajar^1,2,6,#^, Emily S. Wang^1,2,#^, Amy J. Lam^1,2^, Bongjae B. Kim^1,2^, Conor L. Jacobs^3^, Michael W. Davidson^4^, Michael Z. Lin^1,2,5,*^ and Jun Chu^1,2,7,*^

^1^Department of Bioengineering, Stanford University, Stanford, CA, 94305, USA

^2^Department of Pediatrics, Stanford University, Stanford, CA, 94305, USA

^3^Department of Biology, Stanford University, Stanford, CA, 94305, USA

^4^National High Magnetic Field Laboratory, Tallahasee, FL, USA

^5^Department of Neurobiology, Stanford University, Stanford, CA 94305, USA

^6^Current address: Medical Scientist Training Program, University of California at Los Angeles, Los Angeles, CA 90095, USA

^7^Current address: Institute of Biomedical and Health Engineering, Shenzhen Institutes of Advanced Technology, Chinese Academy of Sciences, Shenzhen, 518055, China

#These authors contributed equally to this work

*Correspondence should be addressed to M.Z.L. (mzlin@stanford.edu) or J.C. (jun.chu@siat.ac.cn)


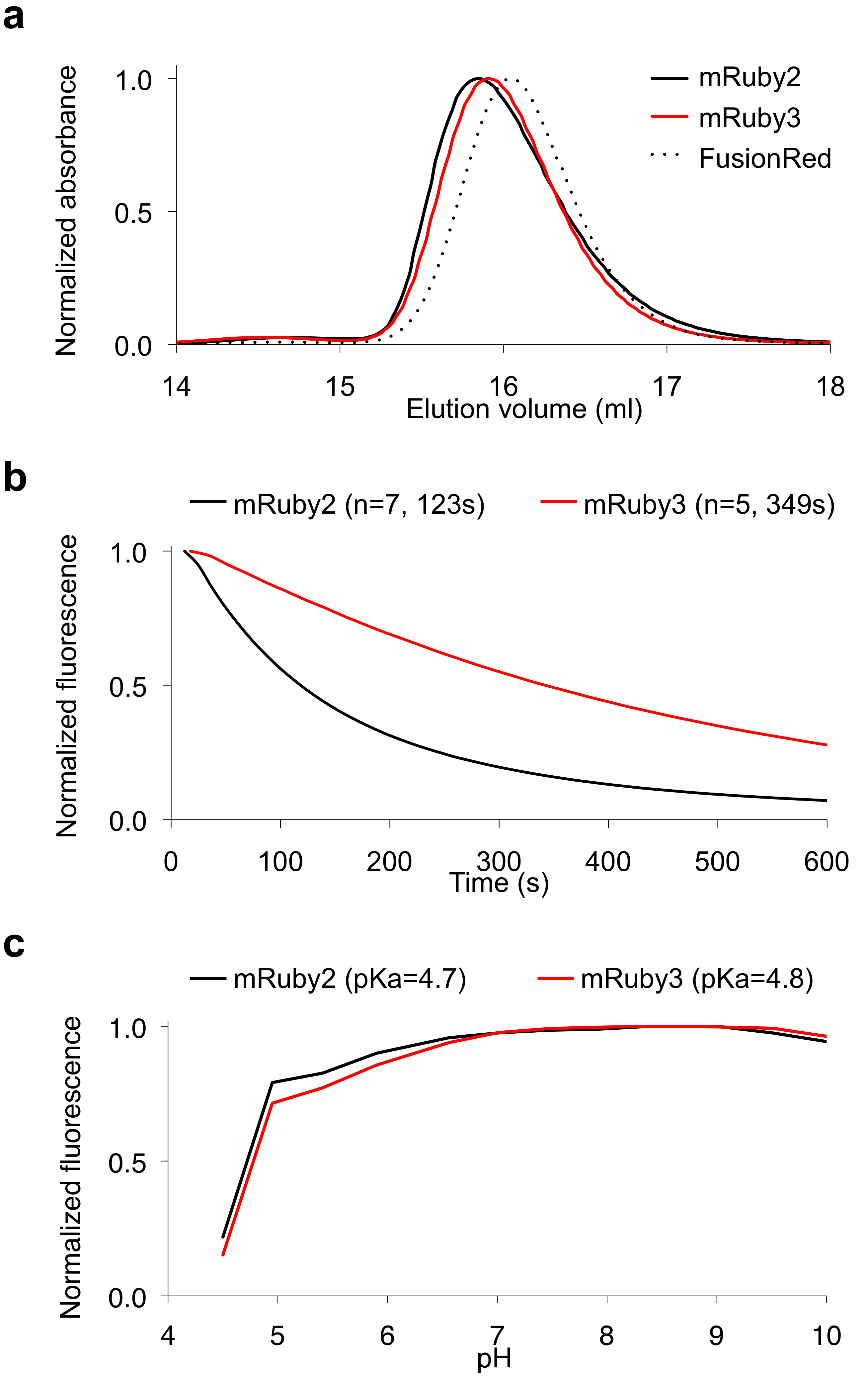


**Supplementary Fig. 1**. *In vitro* characterization of mRuby3. **(a)** Size-exclusion chromatography of the purified mRuby2 and mRuby3 at a concentration of 10 μM. FusionRed is used as a monomeric standard. **(b)** Photobleaching kinetics of purified mRuby2 and mRuby3 under arc lamp illumination with a 545/30 nm filter. **(c)** pH dependence of fluorescence.


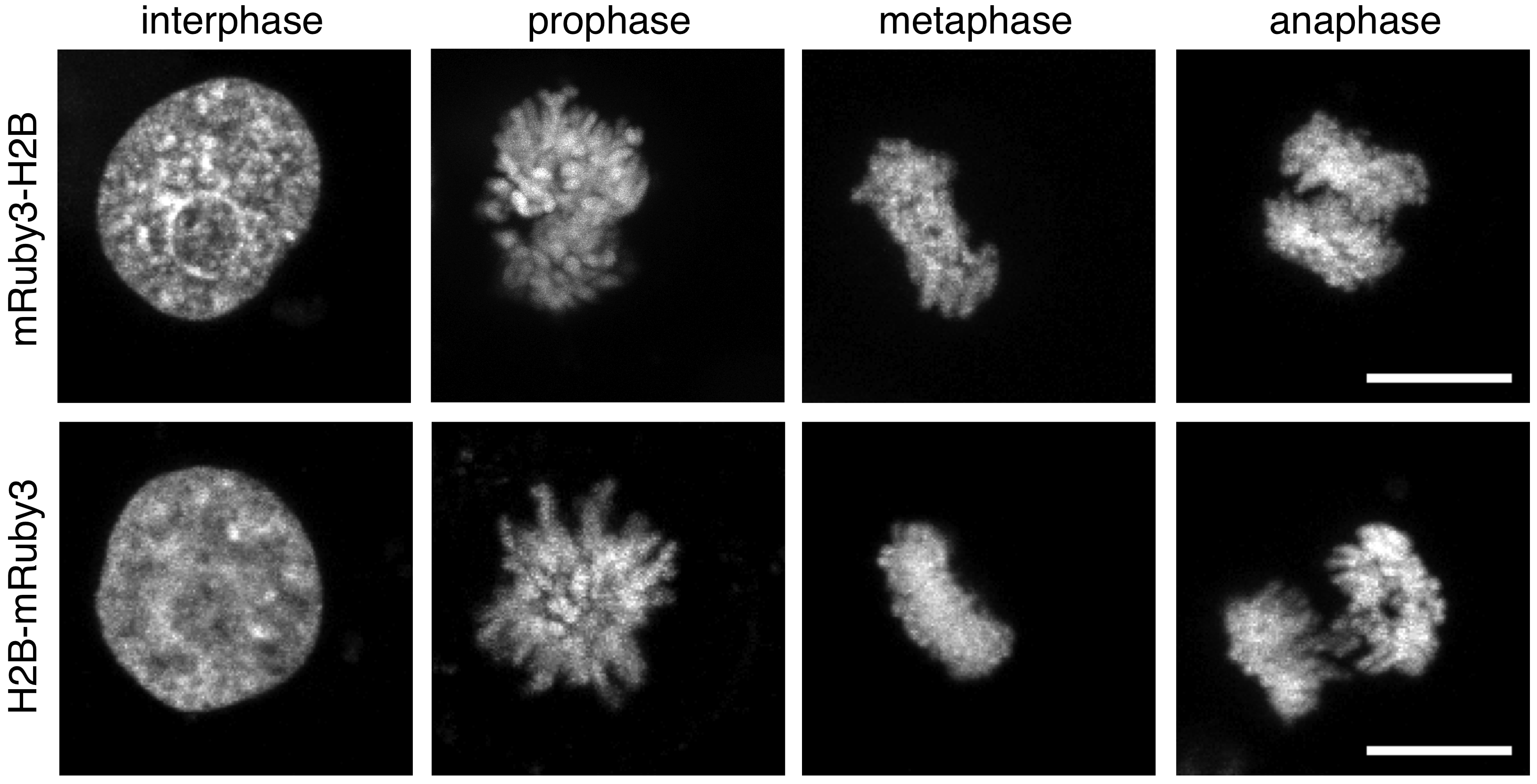


**Supplementary Fig. 2**. mRuby3 permits the visualization of chromatin dynamics during mitosis. HeLa cells express histone H2B fusions with mRuby3 fused at either the N- or C-terminus of H2B (mRuby3-10aa-H2B; H2B-6aa-mRuby3). Linker lengths are denoted by ‘aa’. Panels represent separate cells. Scale bar, 10 µm.


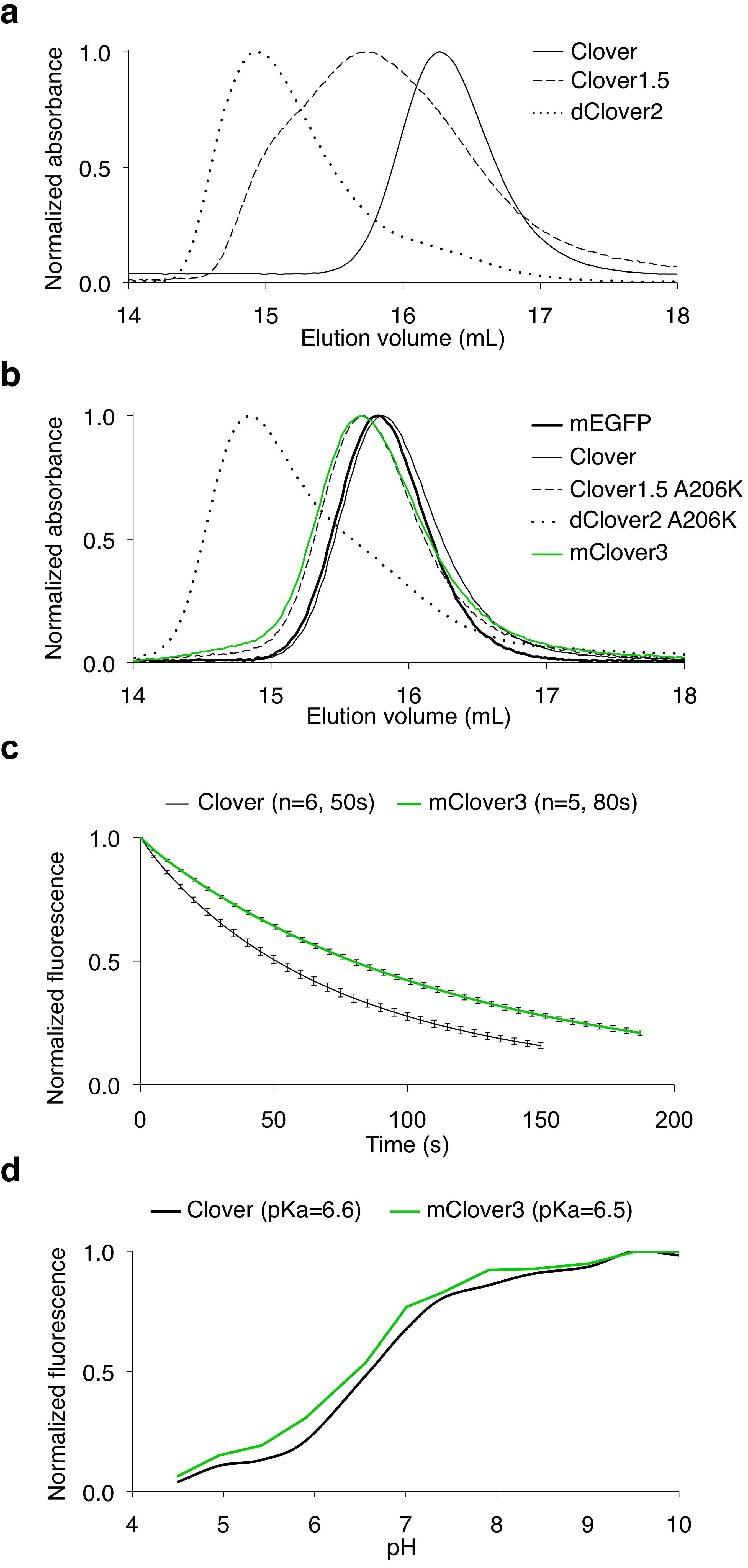


**Supplementary Fig. 3**. *In vitro* characterization of mClover3. **(a,b)** Size-exclusion chromatography of the purified GFPs at a concentration of 10 μM. **(c)** Photobleaching kinetics of purified Clover and mClover3 under arc lamp illumination with a 485/30 nm filter. **(c)** pH dependence of fluorescence.


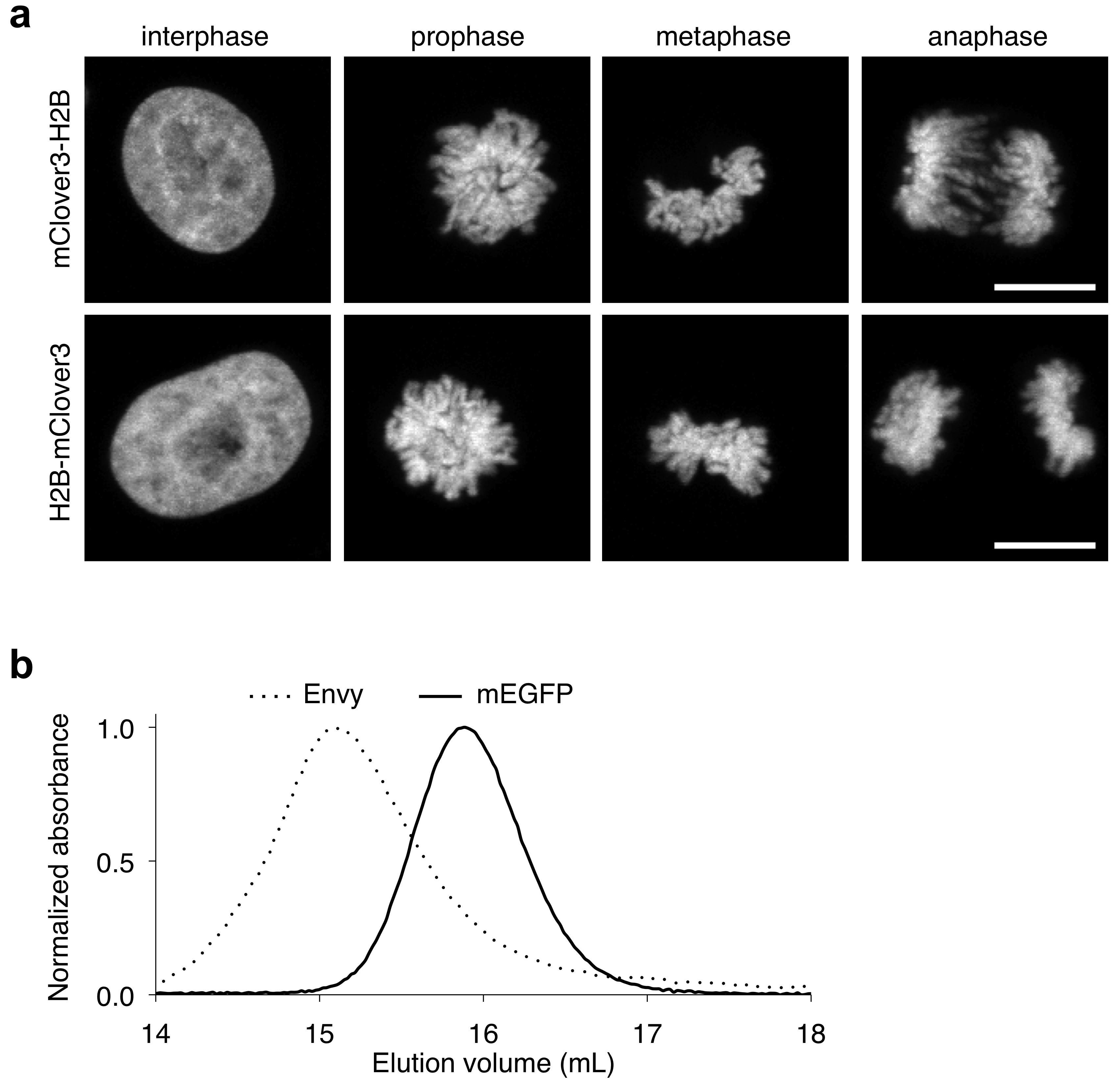


**Supplementary Fig. 4**. (**a**) mClover3 permits the visualization of chromatin dynamics during mitosis. HeLa cells express histone H2B fusions with mClover3 fused at either the N- or C-terminus of H2B (mClover3-10aa-H2B; H2B-6aa-mClover3). Linker lengths are denoted by ‘aa’. Panels represent separate cells. Scale bar, 10 µm. (**b**) Size-exclusion chromatography of purified mEGFP and Envy. 100 μl of purified proteins at 10 μM were used.


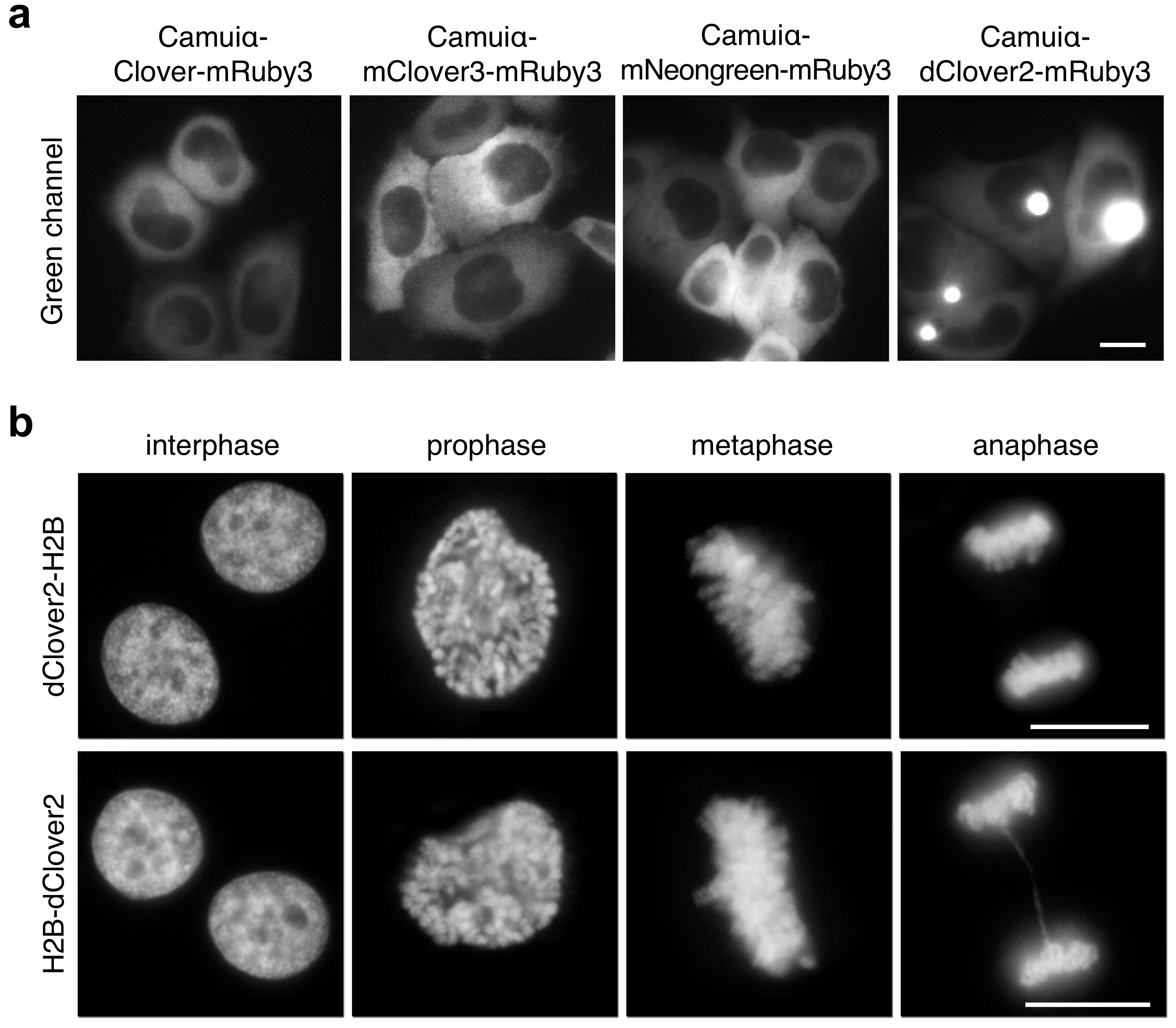


**Supplementary Fig. 5.** Dimericity of green fluorescent proteins interfere with Camuiα expression. (**a**) Camuiα reporter proteins containing Clover or mClover3 are expressed well, but not Camuiα containing dClover2. Scale bar, 10 µm. (**b**) Histone fusions with dClover2 at the N-terminus (top row) or C-terminus (bottom row) do not interfere with mitosis, with cells progressing through interphase, prophase, metaphase, and anaphase. Scale bars, 10 µm.
